# Hydrophobic Eutectogels as Electrodes for Underwater Electromyography Recording

**DOI:** 10.1021/acsmaterialslett.3c00938

**Published:** 2023-11-15

**Authors:** Jon López de Lacalle, Matias L. Picchio, Antonio Dominguez-Alfaro, Ruben Ruiz-Mateos Serrano, Bastien Marchiori, Isabel del Agua, Naroa Lopez-Larrea, Miryam Criado-Gonzalez, George G. Malliaras, David Mecerreyes

**Affiliations:** † POLYMAT University of the Basque Country UPV/EHU, Paseo Manuel de Lardizábal 3, Donostia-San Sebastián 20018, Spain; ‡ Electrical Engineering Division, Department of Engineering, University of Cambridge, 9 JJ Thomson Avenue, Cambridge CB3 0FA, United Kingdom; § Panaxium SAS, Aix-en-Provence 13100, France; ∥ IKERBASQUE, Basque Foundation for Science, Plaza Euskadi 5, Bilbao 48009, Spain

## Abstract

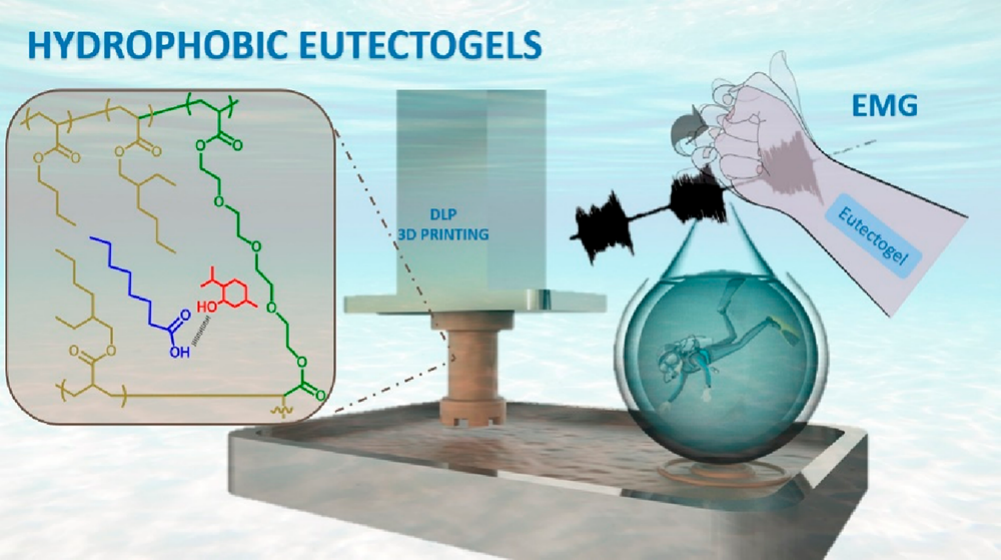

Underwater recording remains a critical challenge in bioelectronics because traditional flexible electrodes can not fulfill essential requirements such as stability and steady conductivity in aquatic environments. Herein, we show the use of elastic gels made of hydrophobic natural eutectic solvents as water-resistant electrodes. These eutectogels are designed with tailorable mechanical properties via one-step photopolymerization of acrylic monomers in different eutectic mixtures composed of fatty acids and menthol. The low viscosity of the eutectics turns the formulations into suitable inks for 3D printing, allowing fast manufacturing of complex objects. Furthermore, the hydrophobic nature of the building blocks endows the eutectogels with excellent stability and low water uptake. The obtained flexible eutectogel electrodes can record real-time electromyography (EMG) signals with low interference in the air and underwater.

Muscle movements and nerve activity in living beings generate bioelectronic currents that can be monitored in real-time, giving vital information for healthcare and medical therapy.^1^ For instance, electromyography (EMG) measures activity in response to muscle activation, which can be used to help detect neuromuscular diseases.^2^ In the past decades, flexible electronic devices have been developed for human biorecording, playing important roles in clinical settings and our daily lives.^3−6^ However, their application is usually limited to air as typical electrodes based on hydrogels or organogels fail in aquatic environments. Unstable signals are often associated with electrode swelling or leakage of organic solvents underwater.^7,8^ Some fluoropolymer-based ionic liquid gels or iongels have been recently proposed to overcome this drawback because they show neglected moisture absorption and can repel water molecules.^9^ For example, Wu and Yu designed water-resistant iongels by free radical polymerization of acryloyloxyethyltrimethylammonium bis(trifluoromethanesulfonyl) imide ([DMAEA-Q][TFSI]) in butyltrimethylammonium bis(trifluoromethanesulfonyl)imide ([N4111][TFSI]). These iongels showed elastomeric behavior, self-healing properties, and stable electrocardiography (ECG) recording in the aquatic environment.^10^ Dong et al. have also investigated the combination of a fluorine-rich ionic liquid monomer, 1-butyl-3-vinylimidazolium bis(trifluoromethanesulfonyl)imide ([BVIm]TFSI), with ethylene glycol methyl ether acrylate and 1-butyl-3-methyl-1H-imidazol-3-ium bis(trifluoromethanesulfonyl)imide ([BMIm]TFSI) ionic liquid to fabricate antiswelling iongels for underwater movement sensors.^11^ However, fluorine- and imidazolium-based ionic liquids are frequently associated with cytotoxic effects and skin irritation, limiting the broad application of these soft ionic materials. Therefore, biosafe soft electrodes that can obtain stable and reliable electrical signals underwater are needed.^12,13^

In this context, natural deep eutectic solvents (NADES) have recently emerged as a new class of green electrolytes sharing many properties of ionic liquids, such as high ionic conductivity, low volatility, and good thermal stability.^14^ Unlike traditional ionic liquids, most NADES are biocompatible, biodegradable, inexpensive, and simpler to manufacture, making them an attractive alternative for bioelectronics.^15^ These solvents are defined as mixtures whose components present enthalpic-driven negative deviations from thermodynamic ideality.^16,17^ These negative deviations are commonly linked to strong interactions between the mixture components, named hydrogen bond acceptor (HBA) and hydrogen bond donor (HBD).^18^ Over the past few years, the available library of NADES has been expanded considerably to incorporate organic acids, sugars, alcohols, polyphenols, terpenes, and terpenoids.^19−22^ Interestingly, some eutectic mixtures are hydrophobic with very low water solubility, opening an unparalleled opportunity for designing biocompatible and cheap soft materials resistant to the aqueous environment.^23^ Among the family of natural hydrophobic eutectic solvents (HES), those based on menthol and fatty acids are being actively studied because of their biocompatibility, low viscosity, and promising bioactive properties.^24−26^ It should be noted that HES based on terpenes and monocarboxylic acids generally exhibits small deviations from ideality. Therefore, although often labeled as NADES, these systems do not present negative deviations large enough to induce a significant melting point depression.^25^ Regardless, their melting points are below room temperature for a wide composition range. Immobilizing these eutectic mixtures into polymer scaffolds would lead to hydrophobic eutectogels that could broaden the landscape of current biorecording.^27^ The concept of eutectogels made of HES is still in an early stage of development, and only a few systems have been reported so far.^28−31^ These materials are mainly based on supramolecular gelators and are unsuitable for electrode fabrication.^32^

Herein, we propose polyacrylate- and menthol-based eutectic formulations as the first example of hydrophobic eutectogels for underwater recording. Eutectic mixtures of this natural terpene and lactic acid (Lac) or fatty acids with increasing chain length, i.e., octanoic acid (Oct), dodecanoic acid (Dodec), and oleic acid (OA) were combined with butyl acrylate (BA), 2-ethylhexyl acrylate (2-EHA), and poly(ethylene glycol) diacrylate (PEGDA, *M*_w_: 700 Da) to obtain flexible eutectogels by one-step free radical photopolymerization (Figure 1A). At this point, it is worth mentioning that, despite being a water-soluble monomer, PEGDA was utterly miscible in all the HES investigated, and it was included in our synthetic blueprint due to its recognized biocompatibility.^33^ The molar compositions of the eutectic solvents are presented in Table S1 of the Supporting Information (SI). All of the eutectic mixtures were liquid at room temperature. FTIR analysis confirmed their formation, which showed a slight bathochromic shift in the carboxylic acid stretching region of the organic acids, probably due to hydrogen bonding interactions with menthol. This slight shift is in line with the findings by Coutinho et al., who demonstrated that the hydrogen-bonding networks established in these mixtures are not significantly different in intensity to those present in the pure compounds.^25^ As an example, the FTIR spectra of Oct:Men (2:1 mol ratio) HES and its pure components are shown in Figure S1.

**Figure 1:**
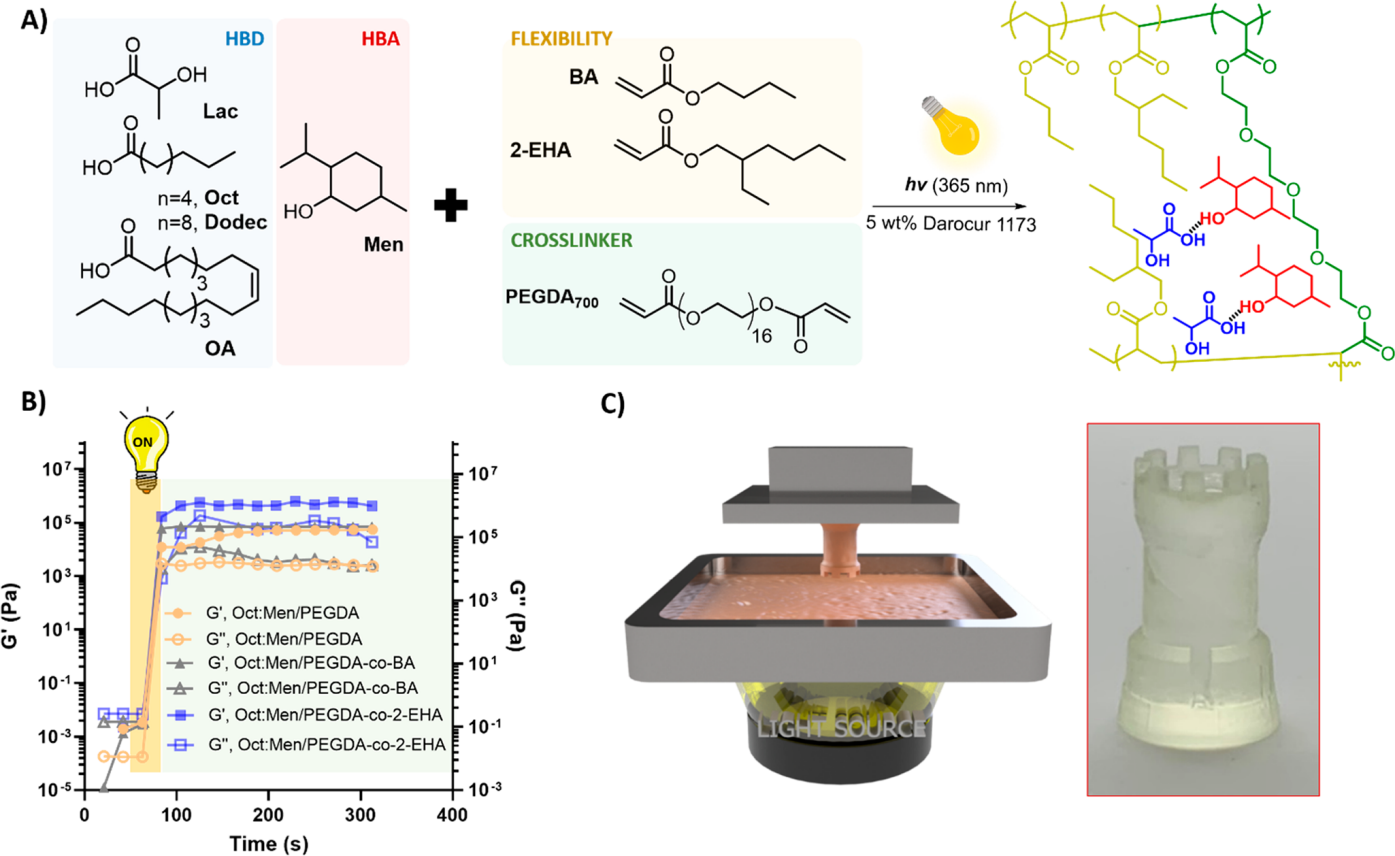
(A) Scheme of the preparation of hydrophobic eutectogels based on organic acid/Men HES and acrylic fomulations. (B) Evolution of elastic (*G*′) and loss (*G*′’) moduli vs. time obtained by photorheology for the main eutectogels formulations. C) Photo of a rook chess piece made of Oct:Men/PEGDA eutectogel obtained by digital light processing 3D printing.

We used photorheology to determine the optimal UV-irradiation time to obtain self-standing eutectogels. For all the formulations, at least 30 s of UV light exposure was needed to achieve an efficient polymerization, resulting in stable viscoelastic solids with storage modulus (*G*′) values higher than viscous modulus (*G*′’), as shown in Figure S2 for Oct:Men/PEGDA eutectogel (60:40 wt %). The photopolymerization kinetics was monitored by FTIR by following the disappearance of the monomer double bond band (810 cm^–1^, C=C out-of-plane bending vibration) while the sample was irradiated with UV light, obtaining full conversion in 1 min (Figure S3).

Besides offering high miscibility in all the HES, PEGDA endowed the materials with good flexibility and high *G*′ values on the order of 10^5^ Pa (Figure 1B and Figure S4). Frequency sweeps revealed that all the HES/acrylic formulations were robust cross-linked gels in the typical 0.1–100 rad/s range (*G*′ ranging 2 × 10^5^-6 × 10^5^ Pa) (Figure S5). We also investigated the incorporation of BA and 2-EHA into the acrylic formulations (HES: PEGDA: BA/2-EHA = 60:30:10 wt %), as these long alkyl chain monomers lead to low glass transition temperature (*T*_g_) polymers that would allow modulating the viscoelasticity of the networks. BA and 2-EHA monomers were miscible in the HES, although their incorporation did not significantly affect *G*′ values after photopolymerization, which remained at around 10^5^ Pa (see Figure 1B for Oct:Men-based formulations).

Interestingly, eutectic mixtures based on menthol and fatty acids featured low viscosity, turning the HES/acrylic formulations into attractive inks for digital light processing 3D printing (DLP). This technique is precious in bioelectronics because it allows fast and cost-effective manufacturing of complex structured electrodes.^34^ Our eutectic formulations allowed the production of 3D structures by DLP in a few minutes with excellent printing fidelity (Figure 1C).

Next, we studied the mechanical properties of the hydrophobic eutectogels by tensile and compression tests. The stress vs. strain curves for Oct:Men HES formulations showed that eutectogels made entirely of PEGDA possess a tensile strength and elongation at breaks of ≈450 kPa and 19%, respectively (Figure 2A). These self-standing eutectogels could be bent and squeezed, showing excellent flexibility (Figure 2B). Furthermore, despite having good miscibility with Oct:Men, the incorporation of BA produced a detriment in the mechanical behavior of the eutectogel, probably due to incompatibilities between the polymer and the eutectic solvent. Conversely, Oct:Men/PEGDA-*co*-2-EHA eutectogels were more stretchable (33% maximum strain) with a good strength of ≈300 kPa. Similar results were observed in compression mode, where Oct:Men/PEGDA-*co*-2-EHA eutectogel outperformed the mechanical parameters of comparable formulations (Figure S6).

**Figure 2:**
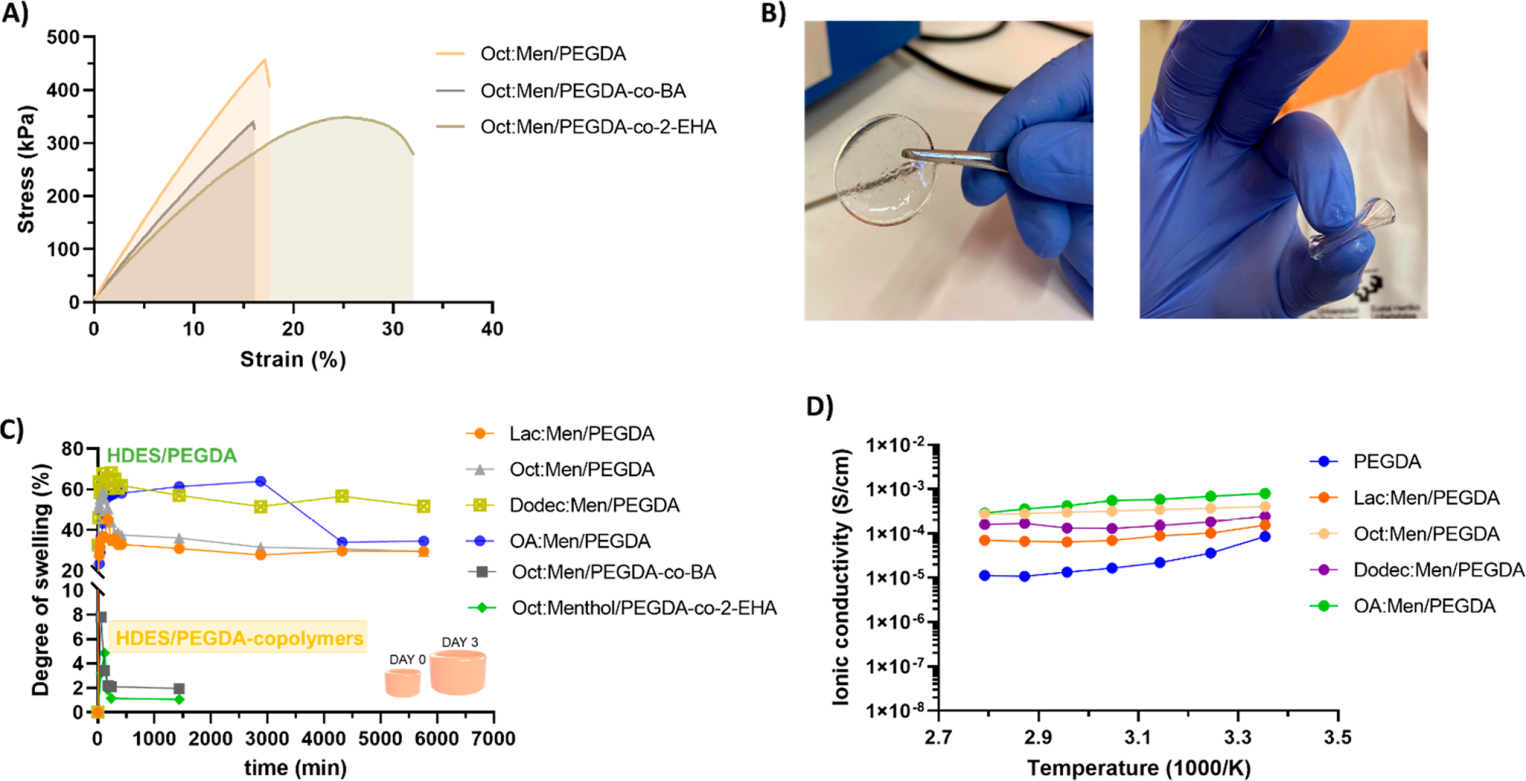
(A) Stress vs. strain curves for eutectogels based on the Oct:Men eutectic mixture. (B) Photos of self-standing Oct:Men/PEGDA eutectogel (left) and when it is compressed and bent (right), showing its flexibility. (C) Swelling curves in a saline buffer for different PEGDA eutectogels, PEGDA-*co*-BA and PEGDA-*co*-2-EHA copolymerized eutectogels. (D) Ionic conductivity of the PEGDA-based hydrophobic eutectogels swollen for 3 days in a saline buffer.

Considering a potential application in underwater recording, high water resistance is a key-sought specification for gel electrodes. Therefore, we evaluated the water uptake of the hydrophobic eutectogels in saline media following their degree of swelling over time (Figure 2C). The maximum water absorption varied with the HES type in PEGDA formulations as follows: Dodec:Men (68%)> OA:Men (64%)> Oct:Men (59%)> Lac: Men (45%). This behavior suggests the formation of gel networks with varied cross-linking densities, probably because of differences in the HES/PEGDA compatibility. However, although less prominent for long-chain acids like Dodec and OA, a small HES leakage was observed, affecting water swelling determination. Notably, the incorporation of BA and 2-EHA in the PEGDA eutectogels significantly improved the water resistance of the eutectogels, reaching maximum swelling of 8% and 5% for Oct:Men/PEGDA-*co*-BA and Oct:Men/PEGDA-*co*-2-EHA, respectively (Figure 2C).

Previous reports have demonstrated that HES change at the dynamic nanoscale when exposed to very low water contents, causing phase segregation and variations in self-diffusion coefficients, viscosity or conductivity.^35^ As a preliminary study to elucidate the optimal HES for underwater recording, we first studied their performance when embedded in a swellable acrylate matrix of PEGDA, mimicking a wet and saline environment. First, the chain length of the HBD was observed to affect the ionic conductivity of the eutectogels. Indeed, impedance spectroscopy analysis (EIS) revealed that PEGDA control displays an ionic conductivity at 25 °C of 8.55 × 10^–5^ S·cm^–1^ while the HES having the shortest chain length, Lac:Men/PEGDA, possesses a slightly higher value of 1.54 × 10^–4^ S·cm^–1^ (Figure 2D). The conductivity is enhanced 1 order of magnitude for longer HBD, up to 7.96 × 10^–4^ S·cm^–1^ for OA:Men/PEGDA, 4.04 × 10^–4^ S·cm^–1^ for Oct:Men/PEGDA, and 2.43 × 10^–4^ for Dodec:Men/PEGDA. If analyzing in detail the impedance at 25 °C, the Nyquist plot can be successfully fitted (0 < χ < 1) in most of the eutectogels to a Randles circuit with a Warburg diffusion impedance in series (Figure S7A). This system is commonly used when a solution or a gel electrolyte is in contact with an electrode. According to Shay and co-workers,^36^ we can assume the resistor in series as the resistance due to the gel (*R*_GEL_) with the electrode, and then the resistor (*R*_CT_) and capacitor (*C*_DL_) correspond to the charge transfer resistance and double layer capacitance created at the interface of an aqueous phase inside of the solid phase. Finally, the Warburg element (Z_W_) refers to the impedance arising from the diffusion of the ions in the gel matrix. Table S2 shows the values for each element fitted. Curiously, PEGDA control and Oct:Men/PEGDA eutectogel present the highest resistance against the electrode (*R*_GEL_), as well as the resistance electrolyte (*R*_CT_) with values of 33 and 621 kΩ and 19 and 183 kΩ for the control and eutectogel, respectively. Indeed, both gels show the lowest values of the capacitance of the double layer (*C*_DL_). Moreover, the semicircle shape of the Nyquist plot of PEGDA control (Figure S7B) and Oct:Men/PEGDA eutectogel (Figure 3A, left) confirms a semi-infinite-length diffusion process.^37^ On the opposite, Lac:Men/PEGDA, Dodec:Men/PEGDA, and OA:Men/PEGDA eutectogels present minimal values of resistance and some orders of magnitude larger C_DL_ if compared with the Oct:Men eutectogel. Furthermore, the Nyquist plot shows finite space diffusion, denoted by a diffusive straight line (Figures S7C, D and 3A, right). It is worth pointing out that these changes in the impedance could be attributed to the structural variation of the polymerized eutectogels, as was highlighted in other works.^38^ However, the disturbance of the hydrogen bonding interactions between menthol and the fatty acid, making the water or other salts act as a second HBD in the eutectogel matrix, could also be a contributing factor.^35^ We hypothesized that the HES could form micelles when the eutectogels are swollen, creating many negatively charged points and giving rise to a double-layer capacitance effect. This behavior is expected to be more remarkable for longer, more lipophilic fatty acids (Figure 3B). The micellization could enhance the ionic conductivity but disrupt the matrix’s continuity, forming phase-segregated domains with a detrimental effect on recording the signal from the muscle to the electrode interface.^39^ To prove our hypothesis, EMG studies were performed on the forearm using a two-electrode configuration (Figure S8A). First, we evaluated the impedance on the skin at 50 Hz, which was similar for all the eutectogels in a dry state (Figure S8B). Interestingly, it dropped 1 order of magnitude after swelling for 3 days in a saline buffer. We know from previous studies that 50 Hz is a crucial frequency to determine differences in the performance of cutaneous electrodes, as it is a midpoint for the clinically relevant bandwidth of EMG (5–400 Hz) biosignals.^40,41^ In this case, all of the electrodes performed equally according to skin impedance values. Second, we tested a forearm movement of extension-contraction, as indicated in Figure S9A, for a whole cycle of swelling, i.e., when the eutectogel was dried and swollen for 1, 2, and 3 days. Moreover, the signal-to-noise (SNR) ratio was calculated for each day (Table S3), considering the signal-to-base values, as indicated in Figure S9B. To obtain more reproducible data, all the measurements of the eutectogels were normalized to the performance of a commercial Ag/AgCl electrode for each day. Figure 3C shows normalized SNR_HES_*/*SNR_Ag/AgCl_ as a function of the swelling time. After 3 days of swelling, long-chain HBD-containing eutectogels, i.e., OA:Men/PEGDA and Dodec:Men/PEGDA, show 18 and 23% decay, respectively, in the SNR value compared with day 2. However, for short-chain HBD, as in Lac:Men/PEGDA, the signal is maintained equally during the 3 days of swelling. It is worth mentioning that the Lac:Men/PEGDA is the only eutectogel that, after 1 day of swelling, lost a 25% SNR, despite subsequently recovering the original SNR quality. This initial decay could be attributed to a partial leakage of the HES due to the water-solubility of Lac. Remarkably, Oct:Men/PEGDA is exclusively improved by 10% compared to day 2 and almost 50% compared to day 1, outperforming substantially the others HES.

**Figure 3:**
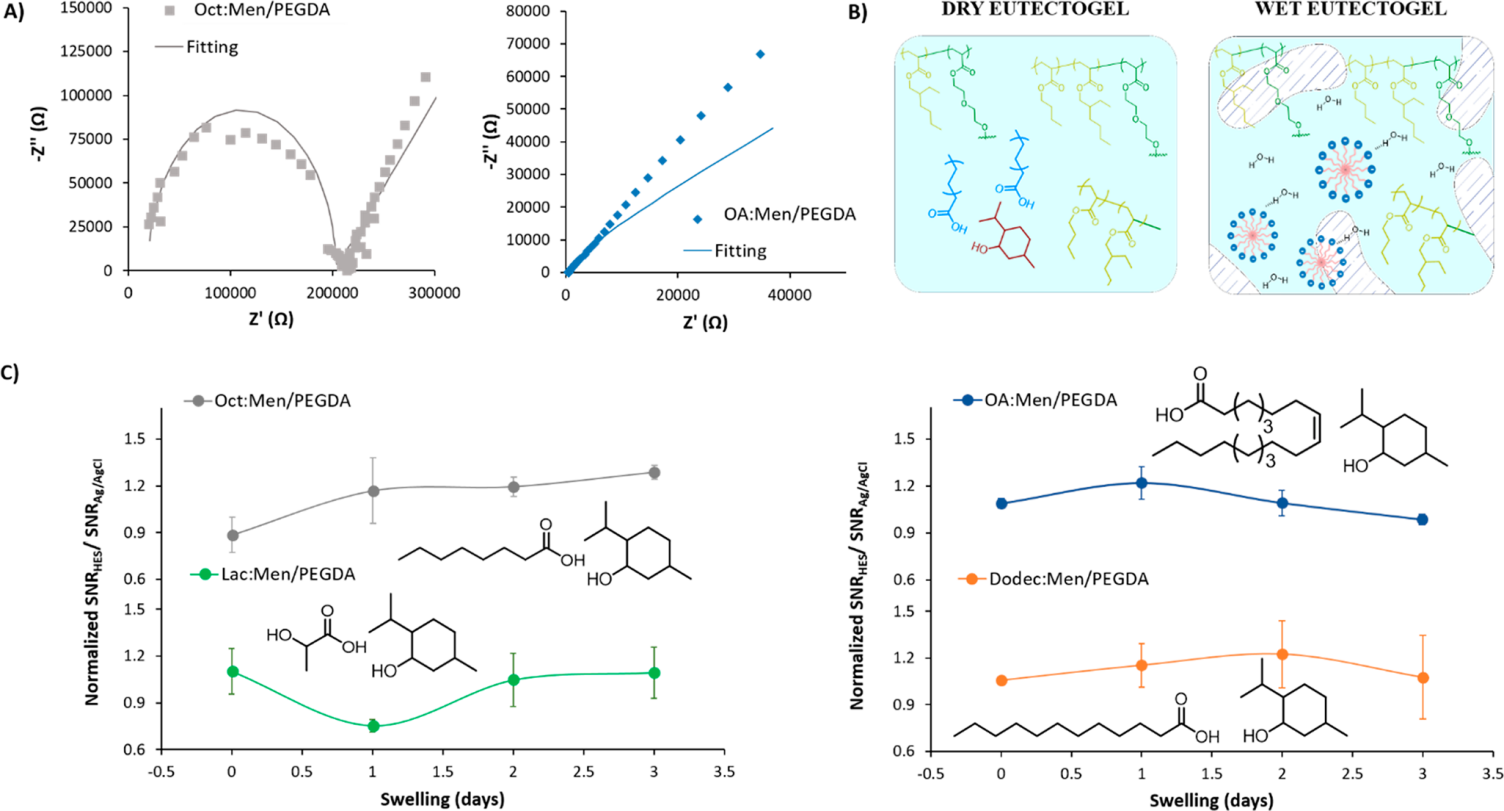
(A) Nyquist plot of Oct:Men/PEGDA (left) and OA:Men/PEGDA (right), obtained by EIS (dot line), and its fitting plot using the equivalent circuit (continuous line). (B) Scheme of the internal structure of the eutectogel in a dry and a swollen state. The HES forms van der Wals interactions between the hydrophobic acrylate matrix and the alkane chain of the HBD. On the opposite side, when the eutectogel is swollen, water makes the HES forms micelles and interrupt the hydrogen bond interaction with menthol, forming phase segregated domains. C) Normalized SNR_HES_/SNR_Ag/AgCl_ of HES/PEGDA eutectogels after 1, 2, and 3 days of swelling in saline buffer.

After evaluating the optimal HES and elucidating the mechanism that commanded the material, we selected a more hydrophobic matrix to embed the eutectic solvent, therefore improving the water insensitivity at the same time as improving stretchability. Hence, 2-EHA-containing formulations, in combination with the Oct:Men HES, turn them into the most promising system for further evaluating their performance in underwater recording. For the underwater EMG recording, electrodes were 3D printed in square shapes of 289 mm^2^, placed in the forearm, and fixed with a wristband. Then, the whole forearm was immersed in a recipient filled with water. Resting and contraction muscle movements were performed and recorded (Figure 4A). It is worth mentioning that the planar gold electrode had the same electroactive area as the printed eutectogels; therefore, its performance is comparable. Figure 4B shows a comparative EMG recording underwater after 3 days of swelling for a long, optimal, and short-chain HBD-composed eutectogel, i.e., OA:Men/PEGDA-*co*-2-EHA, Oct:Men/PEGDA-*co*-2-EHA, and Lac:Men/PEGDA-*co*-2-EHA. Interestingly, Oct:Men/PEGDA-*co*-2-EHA and Lac:Men/PEGDA-*co*-2-EHA eutectogels show clear muscle activation signals and reduced artifacts. Both of them possess similar performance to the bare metal electrode. Conversely, OA:Men/PEGDA-*co*-2-EHA display more artifacts with indistinct signaling, not associated with muscle activation or relaxation. Then, the SNR ratio was calculated and compared after 1 and 3 days of swelling (Figure 4C and Table S4). Lac:Men/PEGDA-*co*-2-EHA (18.57 ± 1.16 dB, day 1, 5.73 ± 3.29 dB, day 3) and Oct:Men/PEGDA-*co*-2-EHA (6.70 ± 1.58 dB day 1, 20.22 ± 3.49 dB, day 3) eutectogels display higher SNR values after 1 and 3 days than both commercial gold electrode (7.45 ± 2.83 dB, day 1, 5.73 ± 3.29 dB, day 3) and OA:Men/PEGDA-*co*-2-EHA (9.72 ± 4.98 dB day 1, 6.39 ± 2.38 dB, day 3), as expected from the above-discussed studies with PEGDA-based eutectogels. A more hydrophobic acrylate matrix could increase the nanophase domains, promoting the micellization effect with more hydrophobic HES. Moreover, Oct:Men/PEGDA-*co*-2-EHA is the only eutectogel that shows an increase above 30% of the SNR performance comparing day 1 against day 3. The results confirm our hypothesis and set eight carbons as the maximum chain length of the fatty acid to obtain hydrophobic electrodes that do not form nanophases without detrimental biorecording potential.

**Figure 4:**
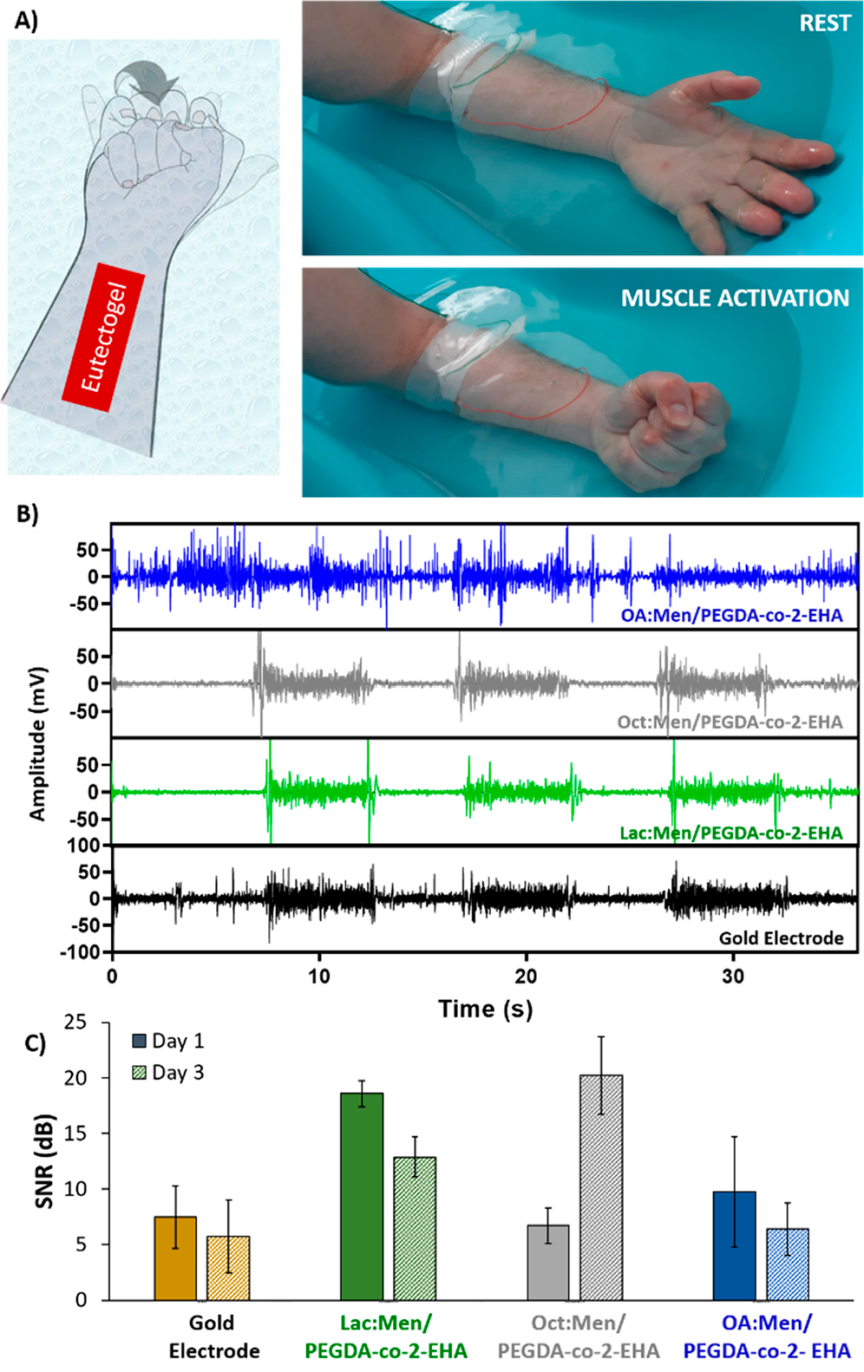
(A) Schematic representation of the position of the electrode on the forearm (left) and photos of the muscle rest and activation in the underwater set up (right). (B) Raw data of EMG recordings on the forearm underwater and (C) SNR of short, optimal, and large HBD-composed eutectogels and its comparative with planar gold electrode.

To conclude, we have demonstrated the use of hydrophobic soft eutectogels based on fatty acids and menthol as electrodes for underwater recording. Hydrophobic eutectogels were obtained by photopolymerization of acrylic monomers within the HES. The mechanical, viscoelastic, and water-swelling behaviors of the eutectogels were investigated and tuned by using different acrylates. Furthermore, the HES/acrylic monomer formulations are suitable inks for 3D printing, allowing for fast manufacturing of complex objects. Among the series of HES explored, long-chain fatty acids, such as Dodec and OA, could undergo a micellization phenomenon when swollen in a saline medium, improving the ionic conductivity of the gels. However, this phase segregation negatively affects the signal recording in the EMG measurements. Interestingly, micellization after swelling seems to not occur in eutectogels based on the Oct:Men solvent, probably because of its shorter aliphatic chain. Therefore, this eutectogel showed the best performance in underwater recording, compared with a standard gold electrode, increasing the SNR after 3 days. Overall, this study represents the first example of biobased and fluorine-free gel electrodes working in an aqueous environment. It is envisioned that hydrophobic eutectogels will open up new perspectives for designing low-cost solid electrolytes for wearable devices and bioelectrodes.

## Supporting Information Available

Materials and methods; chemical structure of HBD and HBA; molar ratio of the as-prepared HES; FTIR spectra of Men, Oct, and Oct:Men; rheological measurements of Oct:Men, Lac:Men, and OA:Men/PEGDA eutectogel; frequency studies of different eutectogels formulations based on PEGDA; compression test of eutectogels based on Oct:Men mixture; swelling curves in a saline buffer for different eutectogels; impedance values obtained on the skin of the different eutectogels; ionic conductivity of eutectogels swollen for 3 days in a saline buffer; SNR of different eutectogels in the dry state and swollen; SNR of different eutectogels recorded in underwater conditions (PDF)

## Abbreviations

HBD: hydrogen bond donor
HBA: hydrogen bond acceptor
NADES: natural deep eutectic solvents
HES: hydrophobic eutectic solvents
Men: menthol
Lac: lactic acid
Oct: octanoic acid
Dodec: dodecanoic acid
OA: oleic acid
PEGDA: poly(ethylene glycol diacrylate)
BA: butyl acrylate
2-EHA: 2-ethylhexyl acrylate
*G*′: elastic modulus
*G*′’: viscous modulus
DLP: digital light processing 3D printing
SNR: signal-to-noise ratios
EMG: electromyography
