# Supplementary material for: Hydrophobic Eutectogels as Electrodes for Underwater Electromyography Recording
Source: ACS Mater Lett. 2023 Nov 15;5(12):3340–6. doi: 10.1021/acsmaterialslett.3c00938 (PMC10698741; doi:10.1021/acsmaterialslett.3c00938)
Supplement: Supplementary file 1 — tz3c00938_si_001.pdf [file tz3c00938_si_001.pdf]

SUPPORTING INFORMATION

Hydrophobic Eutectogels as Electrodes for Underwater Electromyography Recording

Jon López de Lacalle,[1] Matias L. Picchio,[1] Antonio Dominguez-Alfaro,[1,2] Ruben Ruiz-Mateos Serrano,[2] Bastien Marchiori,[3] Isabel del Agua,[3] Naroa Lopez-Larrea,[1] Miryam Criado-Gonzalez,[1] George G. Malliaras,[2] and David Mecerreyes.[1,4]*

[1]POLYMAT University of the Basque Country UPV/EHU, Paseo Manuel de Lardizábal, 3, 20018 Donostia-San Sebastián, Spain. *Email: david.mecerreyes@ehu.es.*

[2]Electrical Engineering Division, Department of Engineering, University of Cambridge, 9 JJ Thomson Ave, Cambridge, UK.

[3] Panaxium SAS, Aix-en-Provence, 13100 France.

[4] IKERBASQUE, Basque Foundation for Science, Bilbao, Spain.

KEYWORDS: Hydrophobic eutectic mixtures, Eutectogels. Bioelectronics, Wearable sensors, Underwater recording.

Experimental

Materials

All chemicals were used without any further purification. Hexanoic acid (Hex, ≥99%), octanoic acid (Oct, ≥98%), dodecanoic acid (Dodec, 98%), poly(ethylene glycol) diacrylate (PEGDA, average M_n 700), butyl acrylate (BA, ≥99%, contains 10-60 ppm monomethyl ether hydroquinone as inhibitor), 2-ethylhexyl acrylate (2-EHA, 98%, contains ≥0.001-≤0.11% monomethyl ether hydroquinone as stabilizer), and 2-hydroxy-2-methylpropiophenone (Darocur 1173, 97%) were purchased from Sigma-Aldrich. DL-menthol (Men, 99 %), DL-lactic acid (Lac, 90%), and oleic acid (OA, 90%) were purchased from Thermo Scientific Chemicals.

HES preparation

Hydrophobic eutectic solvents (HES) were prepared by the heating method, the most commonly used in the literature, based on mixing the two components (menthol and organic acids) and heating them at 65°C under constant stirring until a homogeneous liquid is formed.

Eutectogels synthesis

The HES were mixed with PEGDA (60: 40 wt% ratio) or PEGDA plus an acrylic monomer (BA or 2-EHA) (60: 30: 10 wt% ratio) and the photoinitiator Darocur 1173 (5 wt% based on monomers). The mixture was vortexed for a few seconds, poured into silicone molds, and irradiated using a UVC-5 (DYMAX) UV Curing Conveyor System with a 400 $mW \cdot cm^{-2}$ lamp intensity.

Characterizations

FITR spectroscopy

Fourier transform infrared (FTIR) spectra were recorded on a Bruker Alpha II spectrophotometer employing a Platinum ATR module with a diamond window. All spectra were collected in the range 4000-500 cm^{-1} with a resolution of 4 cm^{-1} and 64 scans.

Rheological measurements

Amplitude and frequency sweep experiments were performed in a stress-controlled Anton Paar Physica MCR101 rheometer, employing an 8 mm parallel-plate geometry. Amplitude sweep experiments were carried out from 0.1 to 25% strain at a constant frequency of 1 Hz at 25 ºC. Frequency experiments were performed from 0.1 to 100 $rad\cdot s^{-1}$ at 1% strain at 25 ºC.

Photorheology

Photorheological measurements were carried out with an AR-G2 rheometer (TA Instruments) using a UV-light lamp (wavelength = 365 nm, power = 2 $mW\cdot cm^{-2}$), oscillation stress of 100 Pa, and 0.1 Hz frequency. A kinetic study was performed to determine the gel point through a continuous registration of the elastic modulus (G′) and loss modulus (G″) before and after UV irradiation. To that aim, the samples were placed on a glass parallel plate of 20 mm diameter and stabilized for 60 s to be subsequently irradiated for 5, 10, and 30 s, and continuing to register G′ and G″ until a plateau was reached.

Kinetic measurement by FTIR

The photopolymerization reaction kinetics was studied by FTIR. Spectra were recorded in attenuated total reflectance (ATR) mode in a Thermo Scientific model Nicolet 6700 FTIR spectrometer, with a resolution of 4 cm^{-1}, mirror speed of 0.3165, and 10 scans. The pre-polymer mixture was placed in a zinc selenide glass, and ATR-FTIR spectra were recorded at room temperature every 0.1 min by exposing the photocurable ink to UV light (wavelength = 390 nm, power = 20 $mW \cdot cm^{-2}$) for 1 min. The conversion was calculated with equation 1 by measuring the area of the peak located at 810 cm^{-1}, which corresponds to the C=C out-of-plane bending vibration:

$$Conversion\ (\%) = (1 - At/A0\ )\ x\ 100 \qquad (1)$$

where At is the area of the band at a time t, and A0 is the area of the band at zero time.

Digital light processing 3D printing

The photopolymerizable eutectogel was placed inside a 3D printing VAT (Asiga Max X35) and exposed to UV light (wavelength = 385 nm, power = 18 $mW \cdot cm^{-2}$). The initial Z-position offset was set at 0.05 mm for the first layer. 3D printing parameters were optimized to 20s of exposure time, layer height of 0.05 mm, and Z-compensation parameter of 0.1 mm. All the structures were designed using Autodesk Inventor 2019 software.

Mechanical test

Gel specimens with bone shapes of 25 mm in length and a cross-section of 3.5 mm × 1 mm were cut for tensile tests. Tests were carried out using a TA HD plus Texture Analyzer equipment (Texture Technologies) at 23 °C, 50% relative humidity, and an

elongation rate of 25 mm·min^{-1}. At least five specimens of each sample were tested. Compression tests were performed on cylindrical samples (10 mm diameter and 1 mm height) at 25 mm·min^{-1} speed and maximum deformation of 70%. All samples were measured in quintuplicate.

Water uptake experiments

For swelling studies, eutectogel discs of 10 mm diameter were immersed in 50 mL of saline buffer (142 mM NaCl, 3 mM $CaCl_2$) at 25 °C. The discs were removed from the saline media at different intervals, superficially dried with tissue paper, weighed, and then returned to the saline buffer container. Then, the degree of swelling (DSt, %) was determined according to Eq. (1), where Wt and Wd are the weight of the swollen and dry eutectogels, respectively. DSt values at each time were plotted for the swelling curves vs. time (min). Measurements were performed in duplicate.

$$DSt = (Wt - Wd)/Wd \times 100 \quad \text{(Eq. 1)}$$

Ionic conductivity measurements

Ionic conductivity (σ) was calculated from electrochemical impedance spectroscopy (EIS) measurements. A symmetrical stainless steel/eutectogel/stainless steel cell was assembled. The distance between the electrodes (l) was kept fixed at ~ 0.543 mm using a silicone spacer ring with an inner area (A) of 0.502 cm^2. EIS measurements were carried out from 85 to 25 °C at every 10 °C intervals performed applying a 10 mV amplitude perturbation in the 1 MHz to 10 mHz frequency range at open circuit potential (OCP) conditions in a potentiostat galvanostat 302N from Autolab coupled to a Microcell HC temperature controller.

The fits of the experimental results were carried out using a nonlinear regression algorithm implemented in the Zview® software. The ohmic resistance (R_b) of the sample at 0.1Hz was used to calculate σ using the following equation:

$$\sigma = \frac{1}{R_b} \frac{l}{A} \quad \text{(Eq. 2)}$$

Fabrication of electrode metal connectors

To fabricate the electrode metal connectors, a Kapton sheet (75 µm) or PET (100 µm) was covered with 10 nm of Ti and 200 nm of gold by thermal evaporation (Alliance Concept). The coated Kapton was laser cut (LPKF protolaser S) with the desired shape. Wires were glued to the gold using a conductive silver paste and then the area was insulated and rigidified using a two parts exoxy resin.

Skin impedance and EMG recording under air conditions

Skin impedances and EMG recording under air conditions were performed after approval of the Ethics Committee of the Department of Engineering at the University of Cambridge (6/9/2018, IONBIKE) and after obtaining informed consent from participants. The measurements were recorded using an RHS stimulation and recording system (Intan Technologies) at a 30 kHz sampling rate. Molded cylindrical eutectogels of 1 cm^2 were used for this experiment. Two electrode configuration was used for this experiment. A commercial Ag/AgCl electrode (MLA 1010B, ADInstruments) was placed on the elbow for voltage reference while the eutectogel was placed on the forearm. For skin impedance or HES/PEGDA EMG evaluation, the cylindrical eutectogels were fixed between the skin and the Au/PET electrodes using a transfer PVA transfer paper (TransOurDream, Glitter

paper). The signal was recorded and filtered by applying a notch filter (50Hz) and band band-pass filter with 10 Hz and 400 Hz cut-off frequencies.

Electromyography (EMG) underwater recording

Electromyography (EMG) recording was recorded on the forearm of a healthy volunteer using a Sienna Ultimate equipment. The sampling frequency was fixed at 1024 Hz, and a hardware notch filter was set at 50 Hz. EMGs were recorded using a plugging of the bottom electrode to the reference. Three electrode configuration was used for this experiment. Two commercial Ag/AgCl electrode (MLA 1010B, ADInstruments) was placed on the elbow and shoulder for voltage reference while the eutectogel was placed on the forearm. For the EMG underwater recordings, Lac:Men/PEGDA-co-2-EHA, Oct:Men/PEGDA-co-2-EHA, and OA:Men/PEGDA-co-2-EHA eutectogels were printed in square shape (289 mm^2) and fixed between the skin and the Au/Kapton electrodes using a wristband. Au/kapton electrodes with the same area were used as control samples. Activation-relaxation experiments on the forearm were performed for this experiment. Firstly, 10 seconds of the base was recorded (noise), then intervals of 5s of muscle activation followed by 5s of rest were recorded. The signal-to-noise ratio was calculated following the next equation:

$$SNR = 20 \cdot \log_{10}\left(\frac{SNR_{signal}}{SNR_{base}}\right) \qquad \text{(Eq. 3)}$$

Finally, all data was processed using a hardware notch filter (50Hz) and band band-pass filter with 10 Hz and 400 Hz cut-off frequencies.

Supporting Information Results

Table S1. Molar ratios of the as-prepared HES.

Hydrogen bond donor (HBD)	Hydrogen bond acceptor (HBA)	Molar ratio
Lactic acid	Menthol	2:1
Octanoic acid	Menthol	2:1
Dodecanoic acid	Menthol	1:2
Oleic acid	Menthol	2:1

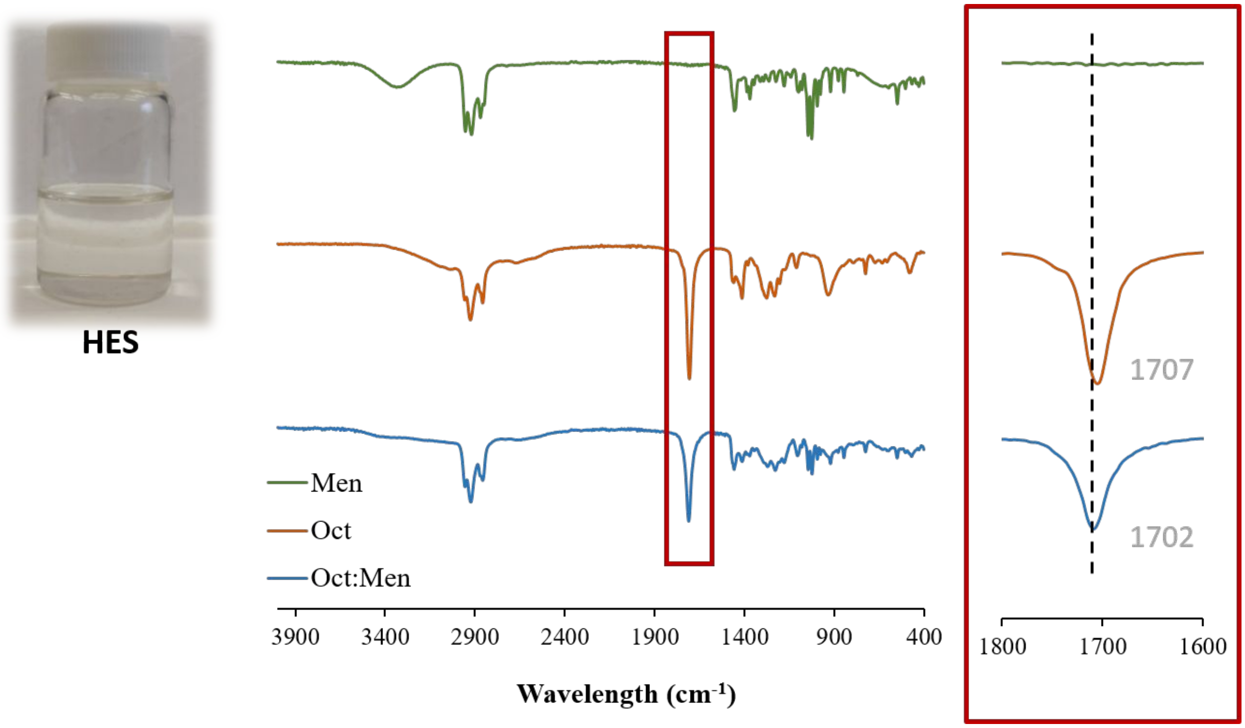

Figure S1. FTIR of Octanoic: Mentol (2:1) HES formed at 60 ºC. Inset: Magnification range from 1800 to 1600 cm^{-1}, indicating the carboxylic acid region (~1700 cm^{-1}). The shifting corresponds to the hydrogen bond formation between menthol and octanoic acid.

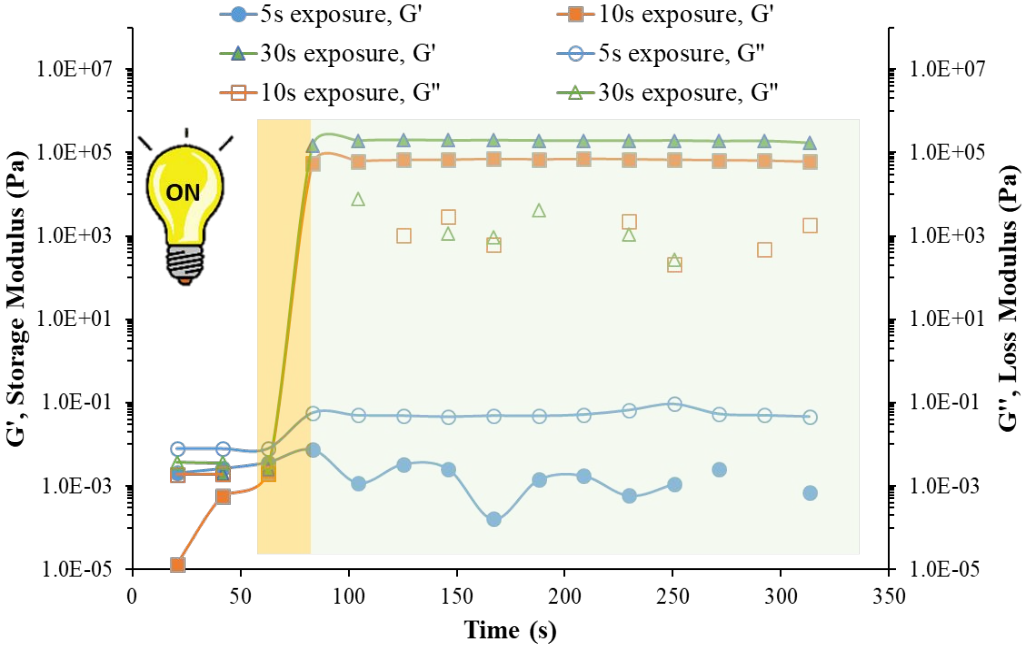

Figure S2. Rheological measurements of Oct: Men/PEGDA, showing the evolution of the elastic modulus (G') and loss modulus (G") over time after different UV-light exposure times.

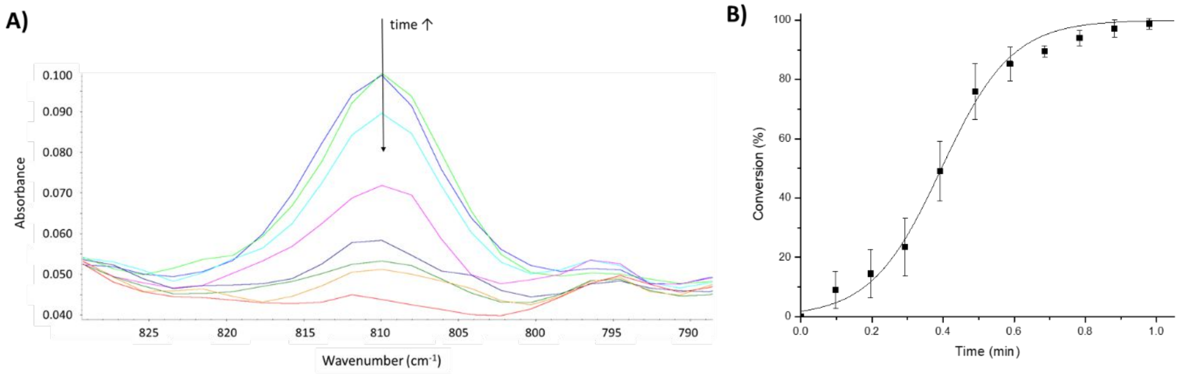

Figure S3. A) Evolution of FTIR C=C out-of-plane bending vibration band (810 cm^{-1}) over time after UV monomer irradiation for Oct:Men/PEGDA eutectogel. B) Progression of monomer conversion calculated from FTIR spectra.

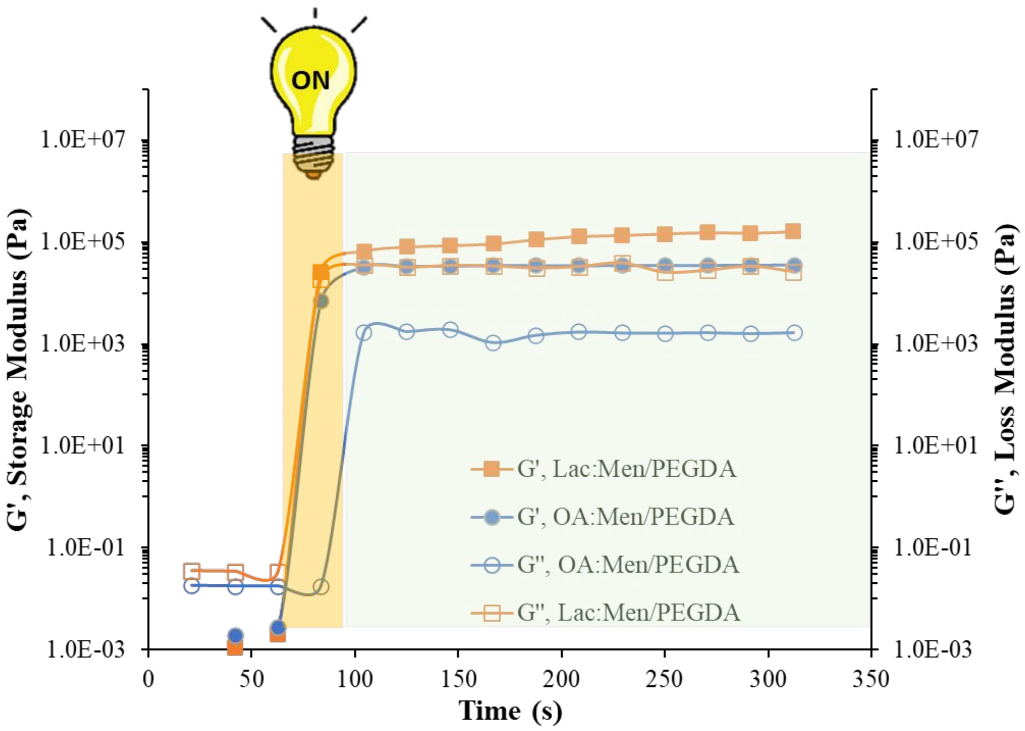

Figure S4. Rheological measurements of Lac: Men and OA: Men/PEGDA eutectogels, showing the evolution of G' and G" after 30 s of exposure to UV light.

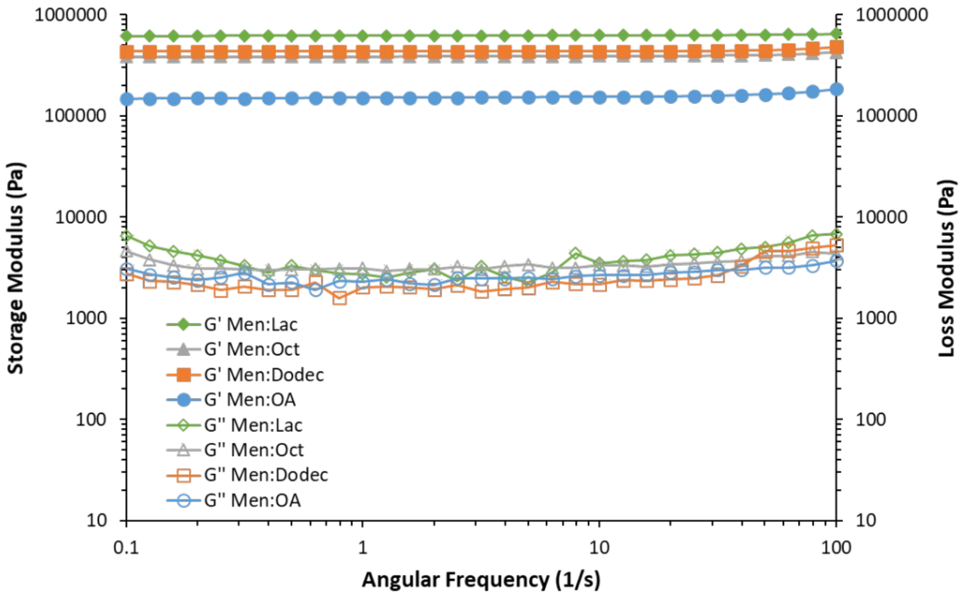

Figure S5. Frequency studies of different eutectogels formulations based on PEGDA in the 0.1-100 rad/sec range.

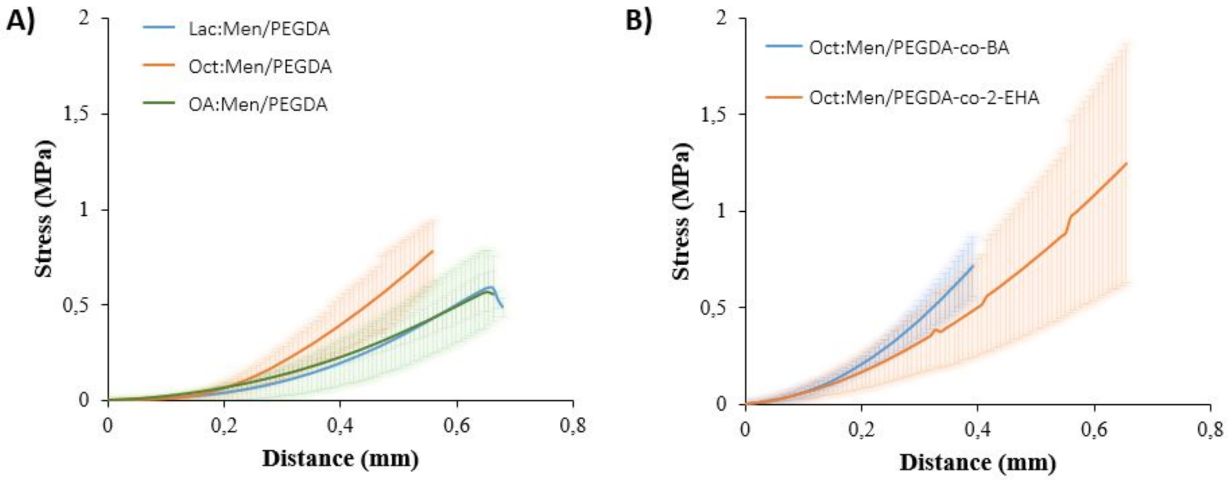

Figure S6. A) Compression test of hydrophobic eutectogels based on PEGDA. B) Compression for eutectogels based on Oct:Men HES.

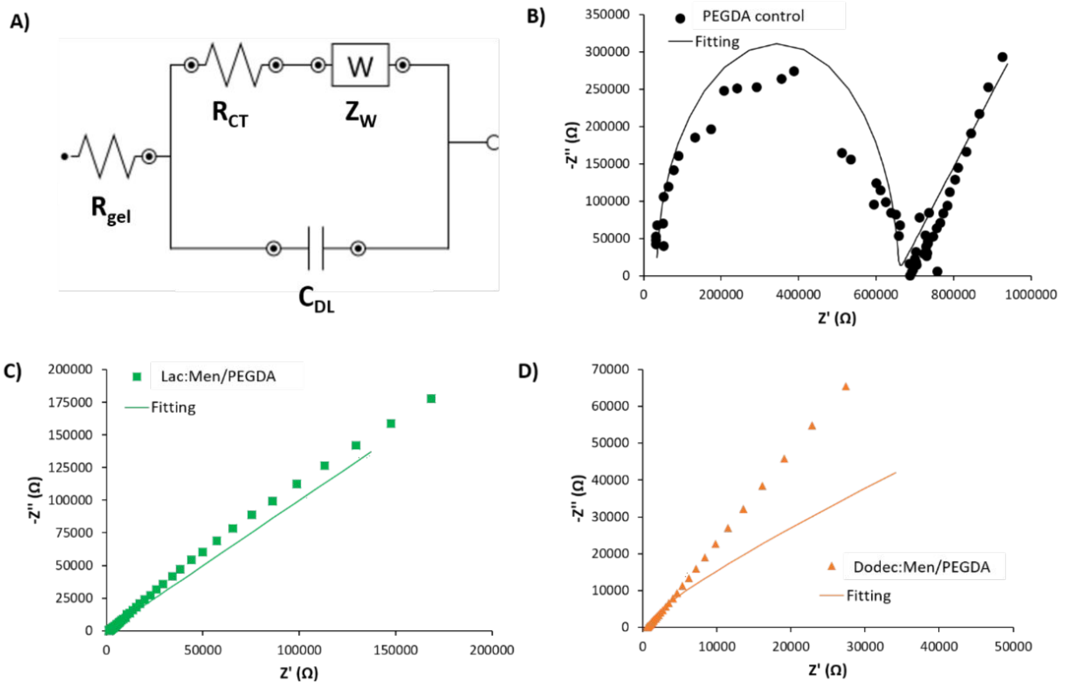

Figure S7. A) Equivalent circuit models of HES/PEGDA, showing the Randles circuit equivalent modified with a Warburg diffusion impedance in series, B-D) Nyquist plot of all the HES/PEGDA obtained by EIS (dot line) and its fitting plot using the equivalent circuit (continuous line).

Table S2. Fitting values obtained of the equivalent circuit

	R_{GEL} (Ω)	R_{CT} (Ω)	C_{DL} (nF)	Z_W	X^2
PEGDA	33600	621000	0.00654	1.81	0.72
Lac:Men/PEGDA	1230	3.63E-05	1.74E+02	3.76	1.07
Oct:Men/PEGDA	19400	183000	0.0093	4.85	0.51
Dodec:Men/PEGDA	603	2.77E-05	1.56E+03	11.9	1.42
OA:Men/PEGDA	502	3.09E-08	1.25E+06	11.4	1.06

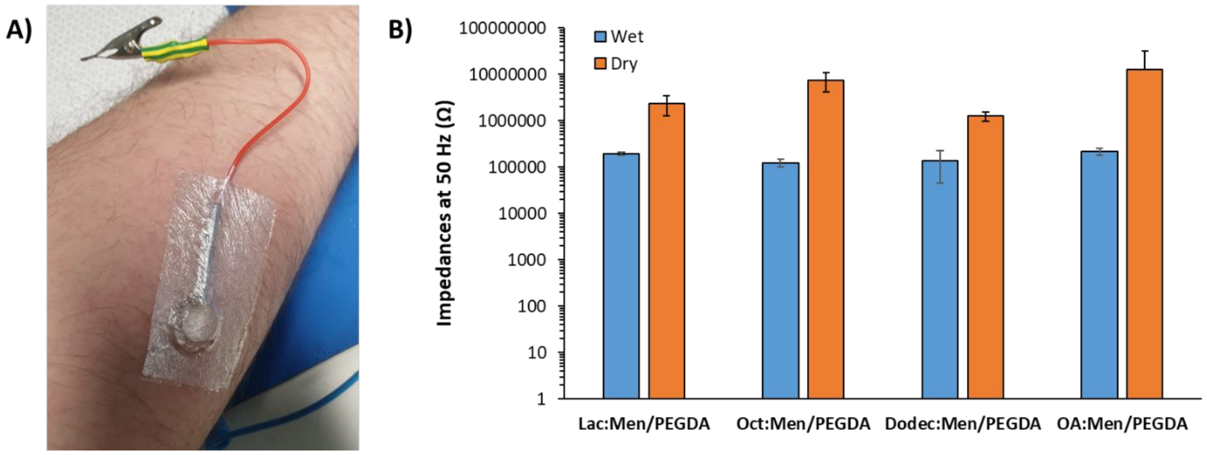

Figure S8. A) Image of a circular electrode placed in the forearm of a male volunteer to measure EIS and EMG and B) Impedance values obtained on the skin of the different hydrophobic eutectogels at 50 Hz.

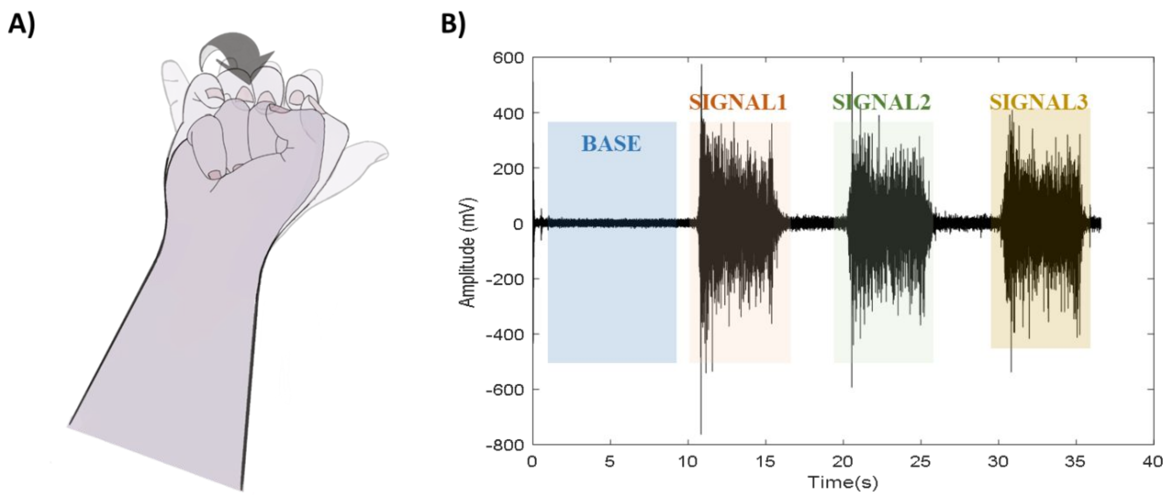

Figure S9. A) Illustration of the EMG experiment used to calculate the signal-to-noise ratio (SNR). The electrode is placed in the forearm, and the hand is closed and open alternatively. B) EMG experiment of Ag/AgCl electrodes on the forearm. The experiment is composed of 10 seconds of base signal and three repetitions of 5 seconds of muscle activation and 5 seconds of rest

Table S3. Signal-to-noise ratios (SNR) of different PEGDA eutectogels in the dry state and swollen after 1, 2, and 3 days in a saline buffer.

	**DRY (dB)**	**SWOLLEN DAY 1 (dB)**	**SWOLLEN DAY 2 (dB)**	**SWOLLEN DAY 3 (dB)**
**AgAgCl**	15.56 ± 2.29	11.22 ± 1.10	13.04 ± 3.87	15.98 ± 4.62
**Lac:Men/PEGDA**	16.57 ± 2.29	8.26 ± 0.43	13.60 ± 2.24	16.30 ± 2.64
**Oct:Men/PEGDA**	13.70 ± 1.74	13.97 ± 2.36	15.36 ± 0.80	20.18 ± 0.71
**Dodec:Men/PEGDA**	16.43 ± 0.22	13.82 ± 1.55	17.18 ± 2.79	17.07 ± 4.32
**OA:Men/PEGDA**	17.18 ± 0.49	13.47 ± 1.17	14.58 ± 1.05	15.49 ± 0.54

Table S4. Signal-to-noise ratios (SNR) of different HES/PEGDA-co-2-EHA eutectogels recorded in underwater conditions after 1 and 3 days of swelling in a saline buffer.

	**SWOLLEN DAY 1 (dB)**	**SWOLLEN DAY 3 (dB)**
**Gold electrode**	7.45 ± 2.83	5.73 ± 3.29
**Lac:Men/PEGDA-co-2-EHA**	18.57 ± 1.16	12.86 ± 1.79
**Oct:Men/PEGDA-co-2-EHA**	6.70 ± 1.58	20.22 ± 3.49
**OA:Men/PEGDA-co-2-EHA**	9.72 ± 4.98	6.39 ± 2.38
